# Transcriptomic Insights into Innate Immunity Responding to Red Rot Disease in Red Alga *Pyropia yezoensis*

**DOI:** 10.3390/ijms20235970

**Published:** 2019-11-27

**Authors:** Lei Tang, Liping Qiu, Cong Liu, Guoying Du, Zhaolan Mo, Xianghai Tang, Yunxiang Mao

**Affiliations:** 1 Key Laboratory of Marine Genetics and Breeding (Ministry of Education), College of Marine Life Sciences, Ocean University of China, Qingdao 266003, China; tangleiouc@163.com (L.T.); qiuliping525@163.com (L.Q.); haiyangliucong@163.com (C.L.); duguo923@ouc.edu.cn (G.D.); Txianghai@ouc.edu.cn (X.T.); 2 Key Laboratory of Maricultural Organism Disease Control, Ministry of Agriculture and Rural Affairs, Yellow Sea Fisheries Research Institute, Chinese Academy of Fishery Sciences, Qingdao 266071, China; 3 Key Laboratory of Utilization and Conservation of Tropical Marine Bioresource (Ministry of Education), College of Fisheries and Life Science, Hainan Tropical Ocean University, Sanya 572022, China

**Keywords:** Transcriptome, *Pyropia yezoensis*, innate immune systems, infection, *Pythium porphyrae*

## Abstract

*Pyropia yezoensis*, one of the most economically important marine algae, suffers from the biotic stress of the oomycete necrotrophic pathogen *Pythium porphyrae*. However, little is known about the molecular defensive mechanisms employed by *Pyr. yezoensis* during the infection process. In the present study, we defined three stages of red rot disease based on histopathological features and photosynthetic physiology. Transcriptomic analysis was carried out at different stages of infection to identify the genes related to the innate immune system in *Pyr. yezoensis*. In total, 2139 up-regulated genes and 1672 down-regulated genes were identified from all the infected groups. Pathogen receptor genes, including three lectin genes (pattern recognition receptors (PRRs)) and five genes encoding typical plant R protein domains (leucine rich repeat (LRR), nucleotide binding site (NBS), or Toll/interleukin-1 receptor (TIR)), were found to be up-regulated after infection. Several defense mechanisms that were typically regarded as PAMP-triggered immunity (PTI) in plants were induced during the infection. These included defensive and protective enzymes, heat shock proteins, secondary metabolites, cellulase, and protease inhibitors. As a part of the effector-triggered immunity (ETI), the expression of genes related to the ubiquitin-proteasome system (UPS) and hypersensitive cell death response (HR) increased significantly during the infection. The current study suggests that, similar to plants, *Pyr. yezoensis* possesses a conserved innate immune system that counters the invasion of necrotrophic pathogen *Pyt. porphyrae*. However, the innate immunity genes of *Pyr. yezoensis* appear to be more ancient in origin compared to those in higher plants.

## 1. Introduction 

Plants suffer from a variety of diseases that are caused by fungi [1,2,3], oomycetes [4,5], bacteria [6], and viruses [7,8,9]. It is evident that plants rely on their innate immune system to resist infection. Jones described the plant innate immune system as a four-phased ‘zigzag’ model [10]. At the onset, plants recognize the pathogen-associated molecular patterns (PAMPs) by transmembrane pattern recognition receptors (PRRs) to activate PAMP-triggered immunity (PTI), which could nonspecifically control the colonization of pathogen. Next, pathogen interferes with the PTI by effectors, resulting in the effector-triggered susceptibility (ETS) of the plant. Further, plants specifically recognize effectors via R proteins directly or indirectly to activate ETI, which could amplify PTI and enhance resistance by activating the hypersensitive cell death in the plant [11]. While the lifestyles of organisms are diverse, the innate immune system exists universally in the vegetable and animal kingdom, with several common features, including PAMP receptors, conserved signaling pathways, and a number of defense mechanisms [12]. In high plants, the innate immune system could be observed in nearly all groups, including moss, non-flowering, and flowering plants [13,14]. However, research on the algae innate immune system is limited and features of the innate immune system in algae have not been described yet.

Similar to plants, algae can potentially get infected by a variety of pathogens [15,16,17]. Several defense mechanisms have been identified in brown algae such as *Ectocarpus siliculosus* and *Saccharina japonica*. *E. siliculosus-Eurychasma dicksonii* has been used as a model of Phaeophyta-oomycete interaction [18,19]. *E. siliculosus* responds to infection by strengthening the cell wall, producing protease inhibitors, reactive oxygen species (ROS), and halogen metabolism [20,21]. *Laminaria digitate*, a model organism in a macroalgae immune study, activates ROS [22,23], releases free fatty acid [24], and employs halogen metabolism and defense gene expression [25] in response to bacterial infection. Another important brown alga, *S. japonica,* could get infected by several alginic acid-decomposing bacterial strains [26]. *S. japonica* generates ROS after the successful recognition of bacterial PAMPs flg22, to fight the pathogen [27]. Furthermore, hypersensitive-programmed cell death (PCD) is observed in *S. japonica* post-infection [27].

*Pyropia yezoensis*, one of the economically important red algae that is massively cultivated in East Asia, is susceptible to *Alternaria* [28], *Olpidiopsis porphyrae* [29], and *Phoma sp.* [30]. Red rot disease, caused by necrotrophic oomycete pathogen *Pythium porphyrae* [31], is lethal to the gametophyte of *Pyr. yezoensis*. The symptoms first appear as red needle-sized lesions on the surface of the thallus, followed by expansion to the whole thallus in two–three days, thus leading to the large-scale necrosis of *Pyr. yezoensis* [32]. Interestingly, few surviving cells surrounded by *Pyt. porphyrae* hyphae could be observed in the lesion [33], which indicates that *Pyr. yezoensis* does possess a defense mechanism to resist the infection. Recently, research discovered that the red rot disease is distributed widely in Western Pacific coastal regions, including China [32], South Korea [34], Japan [35], Philippines [36], and New Zealand [37]. However, little is known about the immune mechanisms in *Pyr. yezonesis* during the infection of red rot disease. It is reported that *Pyropia tenera* could produce reactive oxygen species (ROS), inducing heat shock proteins (HSPs) and cell wall-associated hydrolases to prevent the spread of the oomycete pathogen [38]. In *Pyr. yezoensis*, proteins related to photosynthesis, energy, and carbohydrate metabolism is shown to be suppressed in response to *Pyt. porphyrae* infection [39]. We hypothesize that, similar to plants, *Pyr. yezoensis* might possess an innate immune system to defend against infection. To validate the hypothesis, differentially expressed genes in *Pyr. yezoensis* during *Pyt. porphyrae* infection is obtained by transcriptomic analysis in the current study. Immunity mechanisms in *Pyr. yezoensis* were described and summarized in the current study.

## 2. Results

### 2.1. A Global View of the Transcriptome

**Figure 1:**
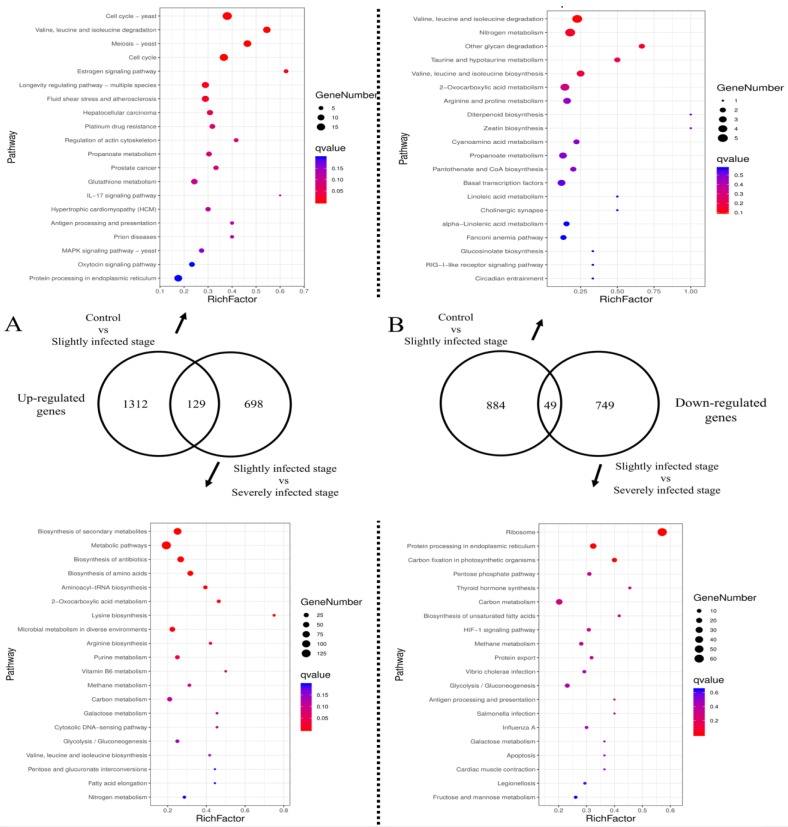
Venn diagram of differential expressed genes and the top 20 pathways of KEGG enrichment. (**A**) Up-regulated genes; (**B**) Down-regulated genes. In KEGG enrichment, “gene number” means the number of *Pyr. yezoensis* genes enriched in this pathway and “rich factor” means the proportion of the number of enriched genes to the total number of genes in this pathway.

In total, 2139 up-regulated genes and 1672 down-regulated genes were identified in the comparison of the slightly infected stage vs. the control group and the severely infected stage vs. the slightly infected stage. As shown in Figure 1, 1312 up-regulated genes were found to be unique to the slightly infected thallus compared with the control group. Further, 698 up-regulated genes were unique in the severely infected stage compared to the slightly infected stage of the thallus. KEGG (Kyoto Encyclopedia of Genes and Genomes) enrichment analysis showed that the function in the two comparisons was different. Unique up-regulated genes in the comparison of the slightly infected stage vs. the control group were mainly related to amino acid metabolism and glycan degradation, but unique up-regulated genes, when the slightly and severely infected stages were compared, were mainly related to the biosynthesis of secondary metabolites. Among the down-regulated genes, 884 genes were unique in the comparison of the healthy and slightly infected stage and 749 genes were unique in the comparison of the slightly and severely infected stage. KEGG enrichment revealed that the function of unique down-regulated genes between the two comparisons was related to several fundamental physiological processes. Unique down-regulated genes were observed when the healthy and slightly infected stages were compared and were mainly related to cell cycle, amino acid degradation, and signaling pathways. Unique down-regulated genes were noted when the slightly and severely infected stages were compared and were mainly related to photosynthesis and protein synthesis.

### 2.2. Potential Pathogen Receptors

#### 2.2.1. Lectins in *Pyr. yezoensis*

PRRs, located on membranes, are the initial pathogen receptors in the innate immune response [10]. Genes containing typical PRR functional domains (LRR, Lectin, EGF, and lysine motif) were searched for by using the Pfam annotation (Protein families, http://pfam.xfam.org/). In *Pyr. yezoensis*, lectin-containing genes and LRR-containing genes were seen, whereas EGF (epidermal growth factor) or lysine motif-containing genes were unable to be found. Among these genes, only lectin-containing genes were predicted as transmembrane proteins by transmembrane region analysis. Transcriptome analysis also showed that the expression of three lectin-containing genes was significantly increased during infection. Compared with the healthy group, these up-regulated genes were specifically expressed in different infected stages: C-type lectin-containing gene *py02002.t1* was only up-regulated in the slightly infected stage by 1.72-fold; two L-type lectin-containing genes *py02275.t1* and *py02843.t1* were only up-regulated in the severely infected stage by 2.36 and 2.44-fold, respectively.

#### 2.2.2. Putative R Proteins

Twenty-three *Pyr. yezoensis* transcripts containing typical R-protein domains such as LRR, NBS, and TIR are summarized in Table 1. Among these genes, only one gene with LRR was up-regulated (FC = 1.89) in the slightly infected stage, three genes with NB-ARC+WD40 domain/motifs and one gene containing the TIR+Pkinase domain were up-regulated by more than 1.5-fold in both the slightly and severely infected stages, and one gene with NB-ARC+TPR was only up-regulated in the severely infected stage (FC = 2.31).

### 2.3. Defense Mechanism in Pyr. yezoensis

#### 2.3.1. ROS-Related Genes

**Figure 2:**
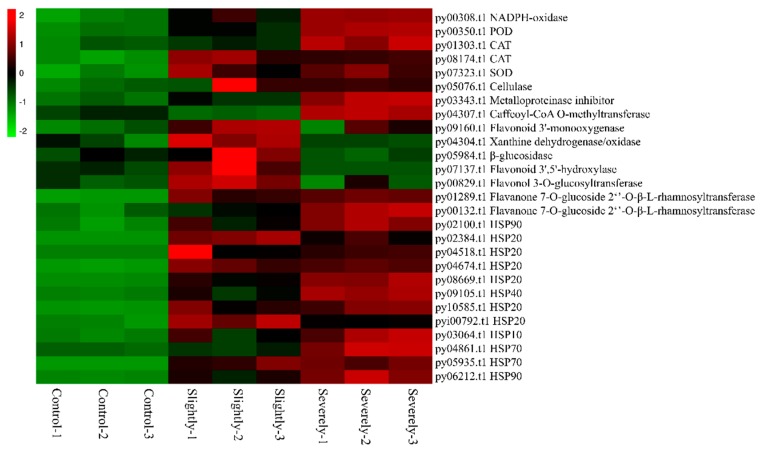
Heatmap of genes related to PTI in *Pyr. yezoensis* during infection. Differential expression is represented in different colors. A negative number indicates down-regulated genes and a positive number represents up-regulated genes. POD: Peroxidase; CAT: Catalase; SOD: Superoxide Dismutase; HSP: Heat shock protein.

The expression pattern of the respiratory burst and cellular anti-oxidation related genes, which includes NADPH-oxidase and antioxidant enzymes, is represented in Figure 2. In *Pyr. yezoensis*, NADPH-oxidase (nicotinamide adenine dinucleotide phosphate oxidase) gene *py00308.t1* up-regulated constantly during the process of infection. The expression of *py00308.t1* increased by 1.68 and 2.36-folds in the slightly and severely infected stages, respectively, compared to that in the healthy group. The up-regulation of antioxidizes, such as peroxidase (POD), catalase (CAT), and superoxide dismutase (SOD), were related to scavenging extra ROS. The expression of POD in *Pyr. yezoensis* increased by 1.6-fold and 2.63-fold during the infection. Two CAT genes were found to be upregulated. CAT-1 was strictly up-regulated in the severely infected stage (FC = 1.87) and CAT-2 was up-regulated in both the slightly (FC = 1.70) and severely infected stages (FC = 1.76). One gene of SOD was up-regulated in both the slightly (FC = 1.60) and severely infected stages (FC = 1.65).

#### 2.3.2. Cellulase

From the transcriptome data of *Pyr. yezoensis*, we identified a cellulase enzyme that could degrade the cellulose, which is the major component of the *Pyt. porphyrae* cell wall. Compared with the healthy *Pyr. yezoensis*, the expression of *py05706.t1* increased by 3.84-fold and 3.57-fold in the slightly and severely infected stages (Figure 2). Bioinformatic analysis showed that py05076.t1 had a signal peptide at the N-terminus and was predicted to be a secretory protein by Target P. It is likely that *Pyr. yezoensis* secreted *py05076.t1* as an extracellular cellulase.

#### 2.3.3. Metalloproteinase Inhibitor

**Figure 3:**
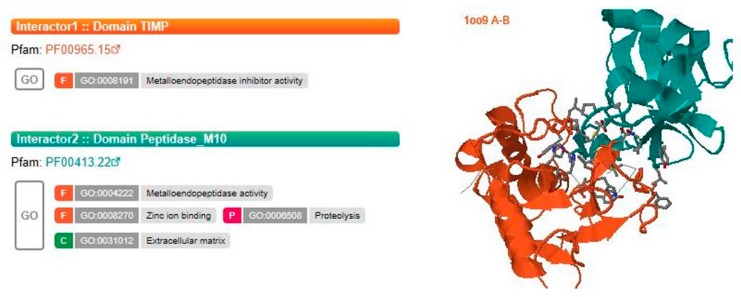
Interaction of *Pyr. yezoensis* metalloproteinase inhibitor and *Pyt. porphyrae* metalloproteinase. The interaction was simulated on database of 3did. Typical domains of the two proteins are shown as interactor1 and interactor2 in the left column. 3D image of interaction of the two proteins is shown in the right column.

The expression of transcript *py03343.t1*, annotated as a metalloproteinase inhibitor, increased by 2.13 and 5.89-folds in the slightly and severely infected stages, respectively, compared to the healthy group (Figure 2). This protein also contained a signal peptide at the N-terminus and was predicted to be a secreted protein. The interaction between the *Pyr. yezoensis* metalloproteinase inhibitor and *Pyt. porphyrae* metalloproteinase was simulated on database of three-dimensional interacting domains (3did, https://3did.irbbarcelona.org) using their protein sequences [40]. As shown in Figure 3, TIMP (tissue inhibitor of metalloproteinase, PF00965), the core domain of *py03343.t1*, could interact with the catalytic center (Peptidase_M10, PF00413) of the metalloproteinase. We speculated that *Pyr. yezoensis* might have secreted *py03343.t1* as an extracellular inhibitor to combine with metalloproteinase in order to restrict the damage from the pathogen.

#### 2.3.4. Secondary Metabolism

**Figure 4:**
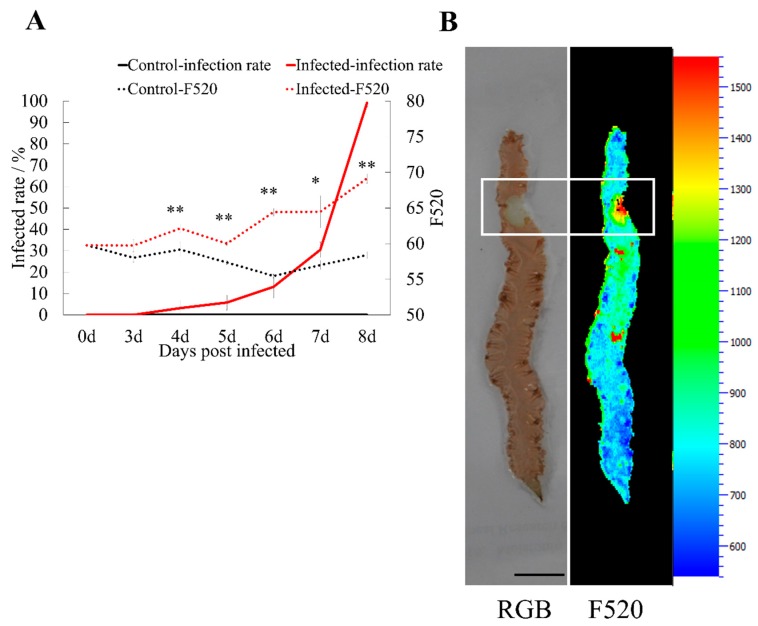
Up-regulated secondary metabolism in *Pyr. yezoensis* after infection (**A**) F520 and infection rate during the infection process. * and ** stands for the significant difference of *p* < 0.05 and 0.01, respectively. (**B**) Secondary metabolism of infected *Pyr. yezoensis* by Multicolor Fluorescence Imaging. RGB: Red, green, blue (RGB) images showing the lesion; F520: Images of green fluorescence (F520). F520 images of the lesion are highlighted by a white rectangle.

F520, a marker for estimating plant secondary metabolite concentration in multi-colour fluorescence imaging (MCFI) was measured during infection. Compared to the control group, F520 intensity of the infected group increased with a significant difference from four-days post-infection when the lesion rate was only 3.7% and increased continuously with the spread of the lesion (Figure 4A). As shown in Figure 4B, fluorescent images of F520 provide a visual representation of the secondary metabolite distribution in different infected stages. F520 increased progressively from the boundary to the center of the lesion. This indicated that *Pyr. yezoensis* could synthesize and accumulate secondary metabolites in response to infection.

The results were further confirmed by transcriptome data. Transcriptome analysis showed that eight genes related to phenolic biosynthesis were up-regulated significantly during infection (FC > 1.5) (Figure 2). The expression pattern of these genes was different between the slightly and severely infected stages. Only three genes were up-regulated in the slightly infected stage, including the luteolin and quercetin synthesis gene *py07137.t1*, isoquercetin synthesis gene *py00829.t1*, and 2-coumaric acid (precursor of coumarin) synthesis gene *py05984.t1*. Only two genes were up-regulated in the severely infected stage—caffeoyl-CoA O-methyltransferase *py04307.t1* and neohesperidin synthesis gene *py00132.t1*. However, another neohesperidin synthesis gene *py01289.t1* was up-regulated in both the slightly and severely infected stages.

#### 2.3.5. Heat Shock Proteins

The transcriptome data also showed that twelve heat shock protein genes (one HSP10, six HSP20s, one HSP40, two HSP70s, and two HSP90s) were up-regulated (FC > 1.5) after infection (Figure 2). Compared to the healthy control group, the expression of these HSP genes was increased significantly in both the slightly and severely infected stages. Among them, HSP20s were the most highly up-regulated heat shock protein groups in response to infection. Five out of six hsp20 genes were up-regulated to more than 15-fold, particularly *py04518.t1* and *py08669.t1*, which were up-regulated to ~200-fold post-infection.

#### 2.3.6. Programmed Cell Death

**Figure 5:**
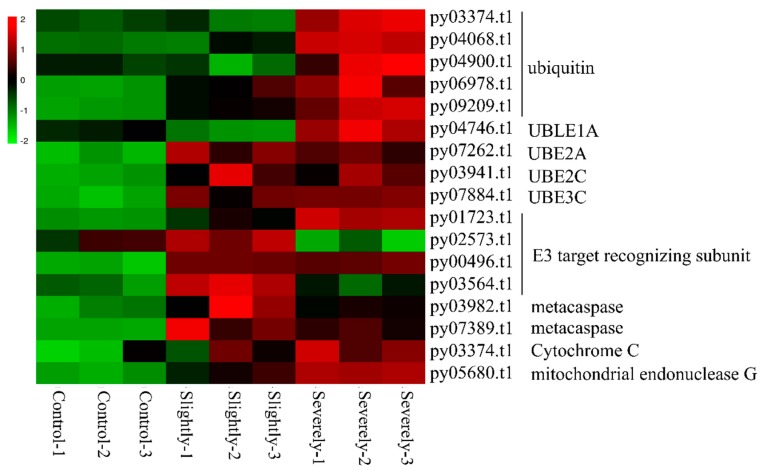
Heatmap of genes related to ubiquitination and programmed cell death in *Pyr. yezoensis* during infection. Differential expression is shown in different colors. A negative number indicates the down-regulation of genes and a positive number indicates the up-regulation of genes. Three biological replicates for control and the slightly infected and severely infected stages are shown.

In plants, hypersensitivity reaction (HR), a form of programmed cell death, is the key disease-resistance mechanism in ETI [41]. Metacaspase, a family of cysteine proteases, are described as the most important regulators of PCD in plants [42]. As shown in Figure 5, the expression of two genes coding for metacaspase were significantly enhanced in the slightly (FC = 2.14) and severely infected stages (FC = 1.84). Cytochrome C, considered the signal of mitochondrial apoptosis, was up-regulated (FC = 2.7) in the severely infected stage. Furthermore, the gene encoding for mitochondrial endonuclease G was induced by 2.04 and 2.84-fold in slightly and severely infected stages, respectively.

#### 2.3.7. Ubiquitination

Ubiquitination plays an essential role in plant PCD regulation. In *Pyr. yezoensis*, all processes related to the ubiquitination system were up-regulated post-*Pyt. porphyrae* infection (Figure 5). In the slightly infected stage, ubiquitin genes, ubiquitin-conjugating enzyme UBE2A, UBE2C, and ubiquitin ligase UBE3C, displayed significant up-regulation. In the severely infected stage, nine genes, which included five ubiquitin genes, ubiquitin-like activating enzyme UBLE1A, ubiquitin-conjugating enzyme UBE2A, UBE2C, and ubiquitin ligase UBE3C, were all up-regulated by more than 1.5-fold.

#### 2.3.8. Epigenetic Modification

**Figure 6:**
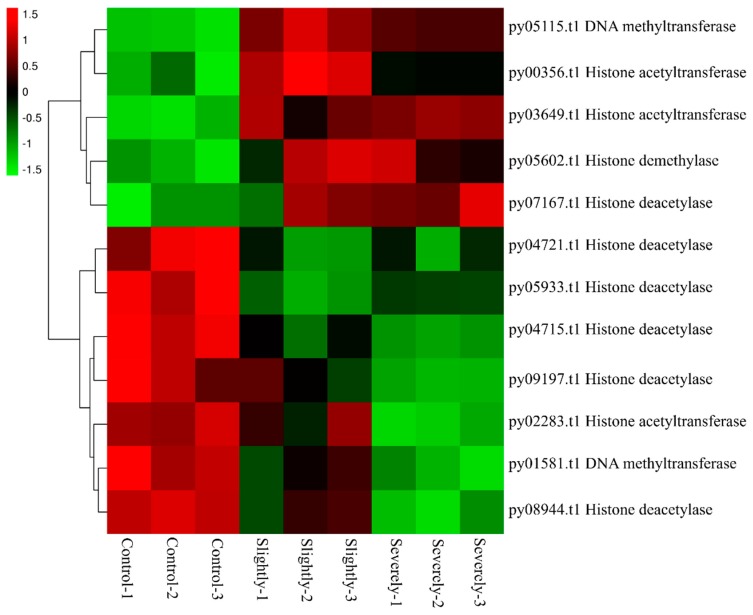
Heatmap of genes related to DNA methylation and histone modification in *Pyr. yezoensis* during infection. Differential expression is shown in different colors. A negative number indicates the down-regulation of genes and a positive number indicates the up-regulation of genes. Three biological replicates for control and the slightly infected and severely infected stages are shown.

It has been proven that plants utilize epigenetic modification to regulate gene expression when exposed to biotic stress [43,44]. In *Pyr. yezoensis*, twelve genes related to DNA methylation, histone acetylation, and histone methylation showed differences expressed after infection of *Pyt. porphyrae*. As shown in Figure 6, five genes, including one DNA methyltransferase, one histone demethylase, one histone deacetylase, and two histone acetyltransferase, were up-regulated significantly (FC > 1.5) at both the slightly and severely infected stages. Meanwhile, seven genes, including five histone deacetylase, one histone acetyltransferase, and one DNA methyltransferase were down-regulated after infection.

## 3. Discussion

### 3.1. Conserved Innate Immune System in Pyr. yezoensis

#### 3.1.1. Sensitive PTI Mechanism

PTI, triggered by pathogenic PAMPs, are the first line of non-specific defense mechanism exhibited by the plant against the pathogen. Several PTI mechanisms have been proven to be sensitive to infection in plants. ROS accumulation occurred at the site of invasion at the initial stages of plant-pathogen interactions [45]. Secondary metabolites and defensive enzymes have been identified as early responses to *Magnaporthe oryzae* in *Oryza sativa* [46]. In *Pyr. yezoensis*, PTI mechanisms, including secondary metabolites, cellulase, metalloproteinase inhibitor, ROS, and HSPs, were up-regulated since the host was exposed to the zoospores at the slightly infected stage. This indicated that the *Pyr. yezoensis* PTI mechanism was extremely sensitive to infection. Successful recognition of PAMPs and PRRs was the first step in the activation of the innate immunity during infection in the plants [10]. Plant lectins played a crucial role in the recognition and binding of carbohydrate PAMPs. Based on the variability in lectin domains, lectin PRRs could be divided into three subclasses: L-type, G-type, and C-type. L-type lectins binded glucose/mannose (Glc/Man) specifically, but the ligand PAMPs of G-type and C-type lectins were still not very evident in the plants [47]. During infection, the *Pyr. yezoensis* C-type lectin gene was only up-regulated in the slightly infected stage and L-type lectin genes were only up-regulated in the severely infected stage. Thus, suggesting that C-type lectin might recognize PAMPs expressed by *Pyt. porphyrae* and then modulate the defense gene expression before direct invasion of the pathogen. L-type lectin might activate or amplify the PTI in the severely infected stage by recognizing glucose and mannose released due to the degradation of the *Pyr. yezoensis* cell wall that consisted of the mannan outer layer and xylan microfibrils in the inner layer [48,49].

It has been reported that several secondary metabolites in red algae *Laurencia majuscule* exhibited antimicrobial activity against “ice–ice” bacteria [50]. In crops, secondary metabolites were frequently used to nondestructively detect diseases using MCFI [51,52]. In the present study, MCFI was first used to measure the concentration of secondary metabolites in diseased algae. The results of F520 indicate that *Pyr. yezoensis* induced secondary metabolite expression before direct invasion of the hyphae, suggesting that *Pyr. yezoensis* secondary metabolism was sensitive to *Pyt. porphyrae* infection and might be used in the nondestructive detection of red rot disease.

Protease inhibitors (PIs), a part of the plant ETI, protect the host by inhibiting pathogen proteases and regulating the activity of host protease [53]. It has been proven that PIs from *Hordeum vulgare*, *Vicia faba,* and *A. thaliana* could inhibit the mycelial growth of several fungal pathogens (broad-spectrum inhibition) [54,55,56]. In the present study, an extracellular metalloproteinase inhibitor was identified for the first time in the algal innate immune system. Consistent with the transcriptomic and proteomic data, this protein expression was up-regulated significantly (FC = 1.52) after infection (further analysis of data in Reference [39]). It appears that *Pyr. yezoensis* secreted the metalloproteinase inhibitor as a “bait” to occupy the metalloproteinase catalytic center and to protect the host protein degradation by pathogen metalloproteinase.

Plants can inhibit the fungal pathogens by secreting chitinase and β-1, 3-glucanase that degrade fungal cell wall during infection [57,58]. In *Pyr. tenera*, several cell-wall associated hydrolases were up-regulated after *Pyt. porphyrae* invasion [38]. In the current study, we analyzed the pathogen cell-wall degrading enzymes in detail. The main constituent of the oomycete cell-wall was β-1,3, β-1,4, and β-1,6-linked glucan skeleton with cellulose and was different from the fungal cell-wall. The *Pyr. yezoensis* cellulase belongs to the glycosyl hydrolase family 5 (cellulase) and was continuously up-regulated during the whole infection process. It might be possible that *Pyr. yezoensis* secreted cellulase to degrade cellulose, thereby inhibiting the formation and expansion of *Pyt. porphyrae* hyphae.

#### 3.1.2. Large HSPs and Single R-Protein Domain-Containing Proteins Might Act as R-Proteins in *Pyr. yezoensis*

R proteins are essential for the plant to activate ETI and create specificity in disease resistance. R proteins with a conserved protein structure of LRR-NBS are extensively present in higher plants. Genes encoding LRR or NBS could also be found in the genome of *Porphyra umbilicalis* [59]. In *Pyr. tenera*, the expression of putative R-proteins showed no significant change during the infection of *Pyt. Porphyrae* [38].Unlike *Pyr. tenera*, six up-regulated putative R-proteins could be observed in *Pyr. yezoensis*. The increase in expression indicated the plausibility of these genes being involved in the *Pyr. yezoensis* innate immunity. However, owing to their simple protein structures, the function of these genes might be limited in the interaction of *Pyr. yezoensis* and *Pyt. porphyrae*.

Several findings demonstrated that large HSPs, such as HSP70s and HSP90s, could recognize pathogen effectors specifically and activate the defense mechanisms against pathogens [60]. Results from a yeast two-hybrid analysis and co-immunoprecipitation indicated that HSP90s might play a role similar to R proteins in plants [61,62]. Considering the absence of NBS-LRR proteins, the up-regulated HSP70s and HSP90s might serve as substitutes for a typical R protein in ETI activation of the *Pyr. yezoensis* innate immune system.

#### 3.1.3. Conversed ETI in *Pyr. yezoensis*

Hypersensitive response (HR), a form of programmed cell death, was triggered after recognition of the R protein and the effectors at the site of infection to prevent the spread of the pathogen [63]. It has been established that metacaspases are the key factors in plant PCD during infection. AtMC1 and AtMC2, two metacaspases in Arabidopsis, possess opposing roles in PCD during *Pseudomonas syringae* infection [64]. However, little is known about the mechanism of PCD in algae during biotic stress. Wang hypothesized that flg22, a typical bacterial PAMP, could induce PCD in female gametophyte of *S. japonica,* depending on the cell ultrastructural changes during infection [27]. It is well known that PCD is more efficient in resisting biotrophic pathogens, but is limited in the defense against necrotrophic pathogens. However, several reports showed that PCD could only inhibit the infection of a few necrotrophic pathogens, such as *Pyrenophora teres* and *Magnaporthe grisea* [65,66]. As a necrotrophic pathogen, *Pyt. porphyrae* could feed on the dead cells or tissues. Nevertheless, PCD related genes that were up-regulated included metacaspase, endonuclease G, and cytochrome C, and these could still be identified in *Pyr. yezoensis* transcripts during infection. These results indicated that PCD was involved in the immune response of *Pyr. yezoensis* against *Pyt. porphyrae*.

In plants, ubiquitin-proteasome system (UPS) was associated with the innate immunity mechanism in a variety of ways, such as PTI modulation, programmed cell death, and signal transduction [67]. E3 was the key enzyme in the process of ubiquitination because it binded to the target protein specifically via its target recognition subunit [68,69]. In *Pyr. yezoensis*, genes related to UPS were up-regulated during infection. Especially in the severely infected stage, all parts of the UPS were up-regulated significantly, indicating that UPS might play a crucial role in *Pyr. yezoensis* to defend against the invasion of *Pyt. porphyrae* hyphae in the severely infected stage.

#### 3.1.4. Overall View of *Pyr. yezoensis* Innate Immune System

**Figure 7:**
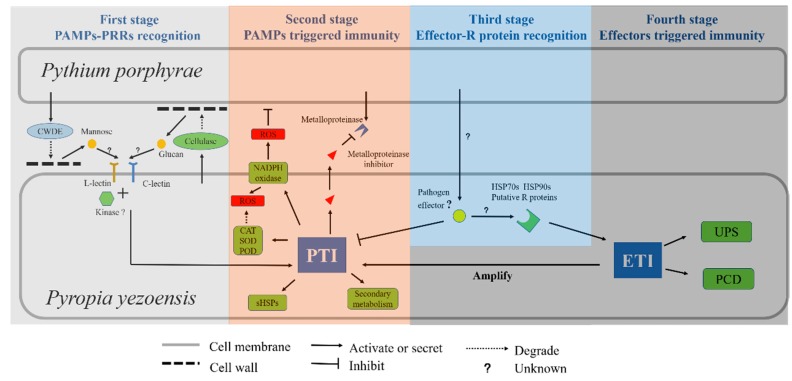
Overall view of the *Pyr. yezoensis* innate immune system during *Pyt. Porphyrae* infection. The entire process of innate immunity in *Pyr. yezoensis* during infection of *Pyt. porphyrae* is depicted. First stage: Carbohydrate PAMPs released during cell-wall degradation is recognized by lectin RLPs. Second stage: With the co-work of kinase, PTI is activated. Third stage: Putative R proteins or large HSPs may recognize the pathogen effectors and activate the ETI. Fourth stage: Genes related to UPS and HR are up-regulated. PTI is also amplified.

While the defense mechanisms in Pyropia have been identified by transcriptomic and proteomics analysis [38,39], the defense responses in different infected stages are still exclusive in *Pyr. yezoensis*. Compared with the innate immune system related genes in plants and algae, we reasonably deduced the overall view of the *Pyr. yezoensis* innate immunity (Figure 7). During the slightly infected stage, *Pyr. yezoensis* cellulase was induced and secreted to degrade the *Pyt. porphyrae* cell-wall. Meanwhile, several types of oligosaccharides were produced that might be recognized as carbohydrate PAMPs by C-type lectins on the host membrane and these worked in concert with an unknown kinase to activate the PTI mechanism. NADPH oxidase was induced to generate ROS. Meanwhile, antioxidase was also up-regulated to overcome oxidative stress. Secondary metabolism, especially the synthesis of phenolics was induced. A metalloproteinase inhibitor was shown to be highly induced and secreted to inhibit pathogen metalloproteinase activity. Several HSP20s, which were involved in damage repair, were also highly up-regulated. In the severely infected stage, hyphae invaded into the cells of *Pyr. yezoensis*. Mannose was released during the cell-wall degradation of *Pyr. yezoensis*. Host L-type lectin recognized the mannose to further activate and amplify the PTI mechanism. Besides this, *Pyr. yezoensis* might rely on ETI to prevent the invasion of a pathogen. In *Pyr. yezoensis*, ETI was activated by potential R-proteins containing the typical R-protein domain. Hsp70s and HSP90s might have played the role of R-proteins to interact with pathogen effectors. Similar to that seen in higher plants, in *Pyr. yezoensis* ETI, the mechanisms described in the PTI were amplified. The ubiquitin system and PCD was also up-regulated to resist the infection.

### 3.2. The Ancient Origin of Genes in the Innate Immune System of Pyr. yezoensis

PRRs are transmembrane proteins with functional ectodomains binding to PAMPs [70]. Based on whether the kinase domain was intracellular in origin or not, PRRs were classified into receptor-like kinases (RLKs) and receptor-like proteins (RLPs) in higher plants [71]. Thus far, all the identified lectin PRRs in plants belonged to RLKs. However, due to the absence of the intracellular kinase domain, lectins in *Pyr. yezoensis* belonged to RLPs. Phylogenetic analysis revealed that the *Pyr. yezoensis* lectin preserved an ancient protein structure and originated earlier than plant lectins (Figures S1 and S2). It has been reported that plant RLPs need an independent kinase to work together [71]. For instance, in tomatoes, Avr9/Cf-9 induced kinase 1 (ACIK1) was required to interact with LRR-RLP *Cf-9* through the assistance of *Cf-9* interacting thioredoxin (CITRX) as a chaperone [72]. Therefore, we surmised that an unknown kinase was required to work with *Pyr. yezoensis* lectin to activate the downstream defense genes.

Nearly all the identified R proteins typically shared commonly conversed domains of TNL (TIR-NBS-LRR) or nTNL (NBS-LRR) [11]. Recent studies analyzed the R protein in plants, Charophytes, Chlorophyta, Rhodophyta, and Glaucophyta [73,74]. Proteins containing NBS or LRR domain were found to exist in all species. However, the fusion events of the NBS and LRR domains were found in Chlorophyta and plants. This indicated that the origin of R protein could be traced back to Chlorophyta, such as *Chromochloris zofingiensis* and *Botryococcus braunii* [74]. In the current study, six up-regulated genes encoding NBS, LRR, or TIR containing proteins were predicted as R protein candidates in *Pyr. yezoensis*. As shown in Figure S3, these genes had ancient origins compared to typical R proteins in plants. Structural analysis showed that all these genes encoded an ancient protein structure of a single R protein domain without fusion events. The similar structures of NBS, LRR, or TIR containing proteins could also be observed in *Porphyra umbilicalis*, such as NBS+TPR and NBS+WD40. The fusion event of NBS and LRR was unable to be found in *Por. Umbilicalis* [59]. The phenomenon of gene fusion was generally found during the evolution of organisms. In archaea and bacteria, the glutamyl and prolyl-tRNA synthetases (GluRS and ProRS) were encoded by two distinct genes. However, a single polypeptide chain protein combining GluRS and ProRS were present in the eukaryotic phylum of coelomate metazoans [75]. Similar co-working mechanisms also existed in *Arabidopsis* R proteins. Due to the lack of LRR, TIR-NBS and TIR-X proteins could interact with NBS-LRR [76]. This suggested that proteins containing a single R protein domain could also act as R proteins with the assistance of other proteins. Therefore, we assumed that single domain containing proteins (NBS, TIR, or LRR) might play the role of the R protein by working in concert.

As shown in Figures S4–S6, the PTI genes of *Pyr. yezoensis*, which included cellulase, metalloproteinase inhibitor and NADH-oxidase, were clustered into a clade of Rhodophyta, which was separate from the clade of plants and Chlorophyta. Therefore, our findings indicated that *Pyr. yezoensis* defense genes preserved the original character in the evolutionary process of the plant innate immune system.

Endosymbiosis drives the evolution and diversification of eukaryotic algae. Plastids originated from cyanobacteria and created three “primary algae”: Glaucophyta, Rhodophyta, and Chlorophyta. Chlorophyta further evolved to land plants [77]. It has been proven that the innate immune system existed in green plants, including mosses and vascular plants [13,14]. Additionally, similar defense mechanisms could be found in algae, which were regarded as the original photosynthetic organisms [20,21,22,23,24,25,26,27]. It indicated that immunity genes might have evolved from homologues that existed in their common ancestors. We speculated that, based on these homologous genes, Pyropia evolved to the innate immune system, which was similar to that in higher plants during the long-term struggle with pathogens independently. Nevertheless, phylogenetic analysis revealed that the defense genes in *Pyr. yezoensis* might have had an ancient origin.

### 3.3. Potential Resistance Mechanisms in Pyr. yezoensis

The innate immune system is considered to be the source of disease resistance in plants, such as non-host resistance and R protein relate resistance [10,78,79]. It has been reported that the early stage response during infection was a key factor in plant resistance regulation [80]. The salicylic acid (SA)-dependent pathway [81], WRKY transcription factor [82], and ROS accumulation [83] have been proven as early responses that contributed to plant resistance against pathogens. In the current study, a number of innate immunity genes at the slightly infected stage were identified in *Pyr. yezoensis*. Therefore, we speculated that, similar to the early defense responses of plants, these early-response genes might potentially be genes related to red rot disease resistance in *Pyr. yezoensis*.

Epigenetic modification plays an important role in plant resistance to fungal and oomycete pathogens. In *A. thaliana*, the hypermethylated mutant was shown to be susceptible to *Hyaloperonospora arabidopsidis* and *Fusarium oxysporum*, while the mutant that DNA methylation establishment impaired was more resistant to both pathogens [84,85]. *A. thaliana* was more susceptible to *H. arabidopsidis* after the mutation of histone lysine methyltransferase ATXR7 [86]. Moreover, epigenetic modification during infection could induce transgenerational defense in plant-pathogen interaction [87,88]. In *Pyr. yezoensis*, a variety of enzymes related to DNA and histone modification were shown to be differentially expressed during red rot disease. We speculated that epigenetic modification was a defense regulation mechanism and might be related to disease resistance development in *Pyr. yezoensis*.

## 4. Materials and Methods

### 4.1. Algae and Pathogen

A lab-cultured pure line RZ of *Pyr. yezoensis* was used as the host in the infection test. Gametophytes were cultured under 40 μmol photons m^−2^ s^−1^ at 10 °C and a photoperiod of light: dark = 12:12 h to obtain thalli. Boiled seawater, supplemented with a Provasoli’s enrichment solution medium (PES) [89], was used as a growth medium.

The *Pyt. porphyrae* strain of NBRC33253 was used as a pathogen in the infection test. The hyphae of NBRC33253 were cultured at 24 °C in a medium of 50% seawater glucose–glutamate medium (SGG) [90]. Zoospores of NBRC33253 were induced, as described previously [91].

### 4.2. Maximum PSII Quantum Yield

The value of the maximum PSII quantum yield was measured by FluorCam 800MF (Photon Systems Instruments, Brno, Czech Republic). Minimal fluorescence (F_0_) was obtained after dark adaption for 15 min. Maximal fluorescence in the dark-adapted state (F_M_) was measured after illuminating it with a saturating pulse (2000μmol photons m^−2^ s^−1^). Maximum PSII quantum yield was calculated using the formula F_V_/F_M_ = (F_M_ − F_0_)/F_M_. F_V_/F_M_ of a different area in the infected thallus was analyzed by FluorCam software7 version 1.2.5.7 on a manual mode. Three biological replicates were performed in each group.

### 4.3. Infection Test and Sampling

Healthy RZ thalli (0.4 g fresh weight) (1.2 ± 0.2 cm in width and 9.3 ± 1.1 cm in length) were exposed to 200 mL zoospore suspension (1 × 10^5^ zoospores mL^−1^) in a flask with PES incubated under 40 μmol photons m^−2^ s^−1^ at 15 °C and a photoperiod of light: dark = 12:12 h for 10 days. Healthy *Pyr. yezoensis* thalli were used as the control group. Three biological replicates were performed in each group.

After lesions appeared, the histopathological characteristics were observed through a microscope. The value of F_V_/F_M_ was measured, as described in the previous step. The infection stages were defined by combining the results of histopathological characteristics and the value of F_V_/F_M_. Briefly, the healthy thalli were defined as a stage of “healthy”; the area beyond the lesion in the infected thallus was defined as a stage of “slightly infected stage” and the lesion area in the infected thallus was defined as a stage of “severely infected stage”. The definition of the infection process was described in detail in the Supplementary Materials: File S2 and Figure S7

The samples of the “slightly infected stage” and “severely infected stage” were separated using a sterile blade under a stereomicroscope. All samples were collected and stored in liquid nitrogen.

### 4.4. RNA Extraction, Construction of cDNA Library, and Sequencing

Nine samples (collected from three groups with three replicates) were ground in liquid nitrogen and a total RNA was extracted using the Plant RNA Kit (Omega Bio-tek, Inc., Norcross, Georgia, USA), following the manufacturer’s instructions. DNase I (Omega) was used to digest the DNA RNA extraction. The quantity and integrity/quality of the total RNA extracted was examined on a Nanodrop and Agilent 2100 bioanalyzer (Agilent Technologies, Inc., Santa Clara, CA, USA).

1μg of total RNA was used to construct a cDNA library using VAHTS™ mRNA-seq V3 Library Prep Kit for Illumina (Vazyme biotech co., ltd., Nanjing, Jiangsu province, China), following the manufacturer’s protocol. High-throughput sequencing was performed on the Illumina HiSeq 2000 platform in a paired-end 150 bp run.

The datasets generated are available in the Sequence Read Archive (SRA) repository accessible through NCBI BioProject ID PRJNA560692 (https://dataview.ncbi.nlm.nih.gov/object/PRJNA560692?reviewer = 99tc7nravboeun8gna76hrjorp).

### 4.5. RNA-seq Analysis

The genome of *Pyr. yezoensis* was was used as a reference genome (DDBJ/ENA/GenBank: WMLA00000000) to filter the reads from infected groups. The genome index was built using Bowtie2 and clean reads were aligned with the genome of *Pyr. yezoensis* using Tophat software. Gene expression was quantified by cuffquant and normalized by cuffnorm to obtain the value of the number of fragments per kilobase per million (FPKM) for each group. Cuffdiff was used to identify differentially expressed genes and the data were manually screened to identify the significantly differentially expressed genes with thresholds of FPKM  >  3 and >1.5-fold (or < 0.67 fold) change. ALL protein sequences used in current study was listed in File S1. 

### 4.6. GO and KEGG Enrichment Analysis

GO enrichment analysis was performed using OmicShare tools, which is a free online platform for data analysis (www.omicshare.com/tools). A KEGG pathway analysis was performed using OmicShare tools (www.omicshare.com/tools).

### 4.7. Prediction of Secretory Proteins

Differentially expressed secretory proteins in *Pyr. yezoensis* were predicted by the following steps [92].

(1) All differentially expressed protein sequences were submitted to SignalP 4.1 online (http://www.cbs.dtu.dk/services/SignalP/) to identify proteins containing a signal peptide [93,94]. Default parameters were used in this experiment.

(2) TMHMM v2.0 (http://www.cbs.dtu.dk/services/TMHMM/) was used to analyze whether transmembrane domains were present in the proteins obtained from SignalP 4.1. Thus, transmembrane proteins were separated from secretory proteins.

(3) Subcellular localization was analyzed by Target P 1.1 Server (http://www.cbs.dtu.dk/services/TargetP/). Proteins localized in mitochondria, chloroplasts, and other subcellular locations were identified and distinguished. Extracellular secretory proteins were obtained for further analysis.

### 4.8. Prediction of LRR, NBS, TIR Domain Containing Genes

HMMsearch was used to predict proteins that had LRR, NBS, and TIR domains in *Pyr. yezoensis*. Seeding sequence alignments of NBS(PF00931), LRR(PF00560), and TIR(PF01582) were generated on the PFAM website (http://pfam.xfam.org) in the Stockholm format. HMMbuild, from HMMER package 3.1b2, was used to create Hidden Markov model (HMM) of the three domains. The profile of HMM was used to search for transcripts with a cut-off E-value of 10^−5^.

### 4.9. Multicolor Fluorescence Imaging

The MCFI of healthy and infected thalli was performed using Multi-color FluorCam (Photon Systems Instruments, Czech Republic). The thallus was tiled with a little boiled seawater in Petri dishes and then kept on the sample table. The excitation wavelength used was 355 nm. The fluorescence spectra of blue (F440), green (F520), red (F690), and far red (F740) were collected. A fluorescence image was displayed with a false-color scale by the FluorCam software7 version 1.2.5.7 (Photon Systems Instruments, Brnocity, Czech Republic). Further, F520 of a different area in the infected thallus was analyzed by FluorCam software7 version 1.2.5.7 on manual mode. Three biological replicates were performed in each group.

### 4.10. Phylogenetic Analysis

Protein sequences of genes related to lectin, R proteins, cellulase, NADPH-oxidaseRBOH, and metalloproteinase inhibitors were collected from GenBank. The GenBank accession number of all the sequences are listed in Table S1. Protein sequences from the current study were used as queries to blast in GenBank using the default parameters. All hits were filtered by employing two criteria: (a) The protein was annotated clearly in the NR or KEGG database; and (b) the protein was from any model plant species and phylogenetically related species. Protein sequences were aligned using CLUSTAL W software and neighbor-joining trees were constructed in MEGA 7.0.

## 5. Conclusions

In the current study, we outlined the defense response of red alga *Pyr. yezoensis* based on the transcriptomic data during the infection of red rot disease. The conserved innate immune system seen in higher plants were also revealed in the red algae. *Pyr. yezoensis* lectins might act as PRRs to activate PTI after the recognition of carbohydrate PAMPs. PTI mechanisms included the up-regulation of ROS, secondary metabolism, metalloproteinase inhibitor, cellulose, and small HSPs in response to infection. PCD and UPS, like ETI, might be activated as a defense mechanism to infection after effector recognition by putative R proteins and HSP70s. Nevertheless, compared to the homologs in higher plants, *Pyr. yezoensis* defense genes appeared to have ancient characteristics. The protein structure of pathogen receptors, which included PRRs and R proteins, were simple with a single functional domain. Therefore, our study provides valuable clues not only for understanding the innate immunity mechanisms of red algae, but also for tracing the origin of the innate immune system in plants. Furthermore, our results provided molecular resources for the breeding of red rot disease resistance strains in *Pyr. yezoensis*, which will continue the sustainable development of nori industries.

## Figures and Tables

**Table 1:** Expression and annotation of putative R proteins in *Pyr. yezoensis.*

Gene ID	Typical Domain	Fold Change (Slightly Infected/Healthy)	Fold Change (Severely Infected/Healthy)	Protein Domains
*py02473.t1*	LRR	1.89 *	0.79	LRR
*py00537.t1*		1.33	1.09	LRR
*py04246.t1*		1.32	1.1	LRR
*py08953.t1*		1.27	1.27	LRR
*py09599.t1*		1.09	0.74	LRR
*py07189.t1*		0.9	0.8	LRR+Hyl III
*py10098.t1*		0.91	1.1	LRR
*py04256.t1*		0.85	0.7	LRR
*py07266.t1*		0.84	0.78	LRR
*py09987.t1*		0.83	1.13	LRR
*py00198.t1*		0.81	1.02	LRR
*py02537.t1*	NBS	2.17 *	1.69 *	NB-ARC+WD40
*py00496.t1*		1.69 *	1.74 *	NB-ARC+WD40
*py07098.t1*		1.64 *	1.52 *	NB-ARC+WD40
*py11015.t1*		1.47	1.38	NB-ARC+TPR
*py07097.t1*		1.37	1.17	NB-ARC+WD40
*py04459.t1*		1.27	2.31 *	NB-ARC+TPR
*py10255.t1*		1.25	1.3	NB-ARC+WD40
*py11066.t1*		1.21	0.75	NB-ARC+WD40
*py07671.t1*		1.11	1.06	Trypsin+NB-ARC+TPR+elF2
*py00971.t1*		1.06	1.24	NB-ARC+TPR
*py04439.t1*		1.05	1.42	NB-ARC+WD40
*py01330.t1*	TIR	1.51 *	1.59 *	TIR+Pkinase
*py08661.t1*		1	1.27	TIR+Pkinase

*: significant change of FMPK (≥1.5-fold or ≤0.67-fold).

## Supplementary Materials

Supplementary materials can be found at https://www.mdpi.com/1422-0067/20/23/5970/s1. Figure S1: Phylogenetic analysis of C-type lectin proteins in *Pyr. yezoensis*. Figure S2: Phylogenetic analysis of L-type lectin proteins in *Pyr. yezoensis*. Figure S3: Phylogenetic analysis of putative R proteins in *Pyr. yezoensis*. Figure S4: Phylogenetic analysis of NADPH oxidase in *Pyr. yezoensis*. Figure S5: Phylogenetic analysis of cellulase in *Pyr. yezoensis*. Figure S6: Phylogenetic analysis of protease inhibitor in *Pyr. yezoensis*. Figure S7: Histopathological features and maximum PSII quantum yield of *Pyr. yezoensis* infected with *Pyt. Porphyrae*. Table S1: Proteins sequences used in phylogenetic analysis. File S1: *Pyropia yezoensis* sequences in current study. File S2: Definition of different infected stages. File S3: Supplementary figure legends

## Abbreviations

PRRs	Pattern recognition receptors
PAMPs	Pathogen-associated molecular patterns
PTI	PAMP-triggered immunity
ETS	Effector-triggered susceptibility
ETI	Effector-triggered immunity
LRR	leucine rich repeat
NBS	Nucleotide binding site
TIR	Toll/interleukin-1 receptor like
UPS	Ubiquitin-proteasome system
HR	Hypersensitive response
ROS	Reactive oxygen species
POD	Peroxidase
CAT	Catalase
SOD	Superoxide Dismutase
HSP	Heat shock protein
FC	Fold change
MCFI	Multi-color fluorescence imaging
