# Supplementary material for: Transcriptomic Insights into Innate Immunity Responding to Red Rot Disease in Red Alga Pyropia yezoensis
Source: Int J Mol Sci. 2019 Nov 27;20(23):5970. doi: 10.3390/ijms20235970 (PMC6928737; doi:10.3390/ijms20235970)
Supplement: Supplementary file 1 [file ijms-20-05970-s001.zip › File S3.pdf]

Fig S1. Phylogenetic analysis of C-type lectin proteins in *Pyr. yezoensis*

The maximum likelihood tree of C-type lectin proteins was constructed based on full-length protein sequences from *Arabidopsis thaliana*, *Oryza sativa*, *Nannoshloropsis gaditana*, *Chlamydomonas reinhardtii*, *Porphyra umbilicalis* and *Pyr. yezoensis*. *Pyr. yezoensis* C-lectin py02002.t1 was highlighted. Corresponding protein structure shown next to the phylogenetic tree. The lines represent the full length of proteins and colored box represent the domains.

Fig S2. Phylogenetic analysis of L-type lectin proteins in *Pyr. yezoensis*

The maximum likelihood tree of L-type lectin proteins was constructed based on full-lenth protein sequences from *Arabidopsis thaliana*, *Oryza sativa*, *Chondrus crispus*, *Porphyra umbilicalis*, *Ectocarpus siliculosus* and *Pyr. yezoensis*. *Pyr. yezoensis* L-lectin was highlighted. Corresponding protein structure shown next to the phylogenetic tree. The lines represent the full length of proteins and colored box represent the domains.

Fig S3. Phylogenetic analysis of putative R proteins in *Pyr. yezoensis*

The maximum likelihood tree of putative R proteins was constructed based on full-lenth protein sequences from *Arabidopsis thaliana, Chromochloris zofingiensis, Porphyra umbilicalis, Ectocarpus siliculosus* and *Pyr. yezoensis*. *Pyr. yezoensis* putative R proteins was highlighted. Corresponding protein structure shown next to the phylogenetic tree. The lines represent the full length of proteins and colored box represent the domains.

Fig S4. Phylogenetic analysis of NADPH oxidase in *Pyr. yezoensis*

The maximum likelihood tree of NADPH oxidase was constructed based on full-lenth protein sequences from *Arabidopsis thaliana*, *Zea mays, Oryza sativa, Chlamydomonas reinhardtii*, *Chondrus crispus*, *Porphyra umbilicalis*, *Phaeodactylum tricornutum* and *Pyr. yezoensis*. *Pyr. yezoensis* NADPH oxidase py00308.t1 was highlighted.

Fig S5. Phylogenetic analysis of cellulase in *Pyr. yezoensis*

The maximum likelihood tree of cellulase was constructed based on full-lenth protein sequences from *Arabidopsis thaliana, Oryza sativa, Zea mays, Monoraphidium neglectum, Micromonas commoda, Chondrus crispus, Gracilariopsis chorda* and *Pyr. yezoensis*. *Pyr. yezoensis* cellulase py05067.t1 was highlighted.

Fig S6. Phylogenetic analysis of protease inhibitor in *Pyr. yezoensis*

The maximum likelihood tree of cellulase was constructed based on full-lenth protein sequences from *Arabidopsis thaliana*, *Oryza sativa*, *Chromochloris zofingiensis*, Chlorella variabilis, *Volvox carteri*, *Chondrus crispus*, *Porphyra umbilicalis* and *Pyr. yezoensis*. *Pyr. yezoensis* cellulase py03343.t1 was highlighted.
