# Supplementary material for: Transcriptomic Insights into Innate Immunity Responding to Red Rot Disease in Red Alga Pyropia yezoensis
Source: Int J Mol Sci. 2019 Nov 27;20(23):5970. doi: 10.3390/ijms20235970 (PMC6928737; doi:10.3390/ijms20235970)
Supplement: Supplementary file 1 [file ijms-20-05970-s001.zip › Files S2.pdf]

Definition of different infected stages

As shown in Fig.S7 A&A1, the cells in healthy thallus showed complete cell shape with clear chromatophore and vacuoles. Compared to healthy thallus, the infected thallus could be easily divided into two parts: lesion and area beyond lesion (Fig. S7 B). In the lesion area, the hyphae of *Pyt. Porphyrae*(Fig. S7 B2, arrow2) grew profusely and penetrated into the *Pyr. yezoensis* cells. Due to the infection, the host cells began to turn to deep purple-colored and atrophied (Fig. S7 B2, arrow1). A few cells discharged peach-colored pigments. However, the cells in the area beyond lesion showed no morphological differences from cells in the healthy thallus. Histopathological observation could not distinguish the cells in the area beyond the lesion of the infected thallus and cells in the healthy thallus.

However, according to the experimental design, there were about 3×10^3 zoospores distributed per square millimeter of the *Pyr. yezoensis* thalli. Therefore, we inferred that the zoospores might get attached to the surface of *Pyr. yezoensis* and cause changes in the host physiological state. We introduced the chlorophyll fluorescence techniques to test photosynthetic characteristics during infection. As shown in Fig.1 C, compared to healthy thallus (Fv/Fm= 0.45±0.014), Fv/Fm of the area beyond the lesion in the infected thallus declined to 0.35±0.014 significantly. Similarly, the Fv/Fm of lesion area declined to 0.24±0.014 significantly compared to the healthy thallus. The Fv/Fm of slightly and severely infected stages decreased on an

average to 22.2% and 46.7% respectively. This indicates that although the cells have not been invaded by hyphae, the metabolism has been affected by the infection.

Hence, we take the results of chlorophyll fluorescence as a supplement to the results of histopathological observation and used both parameters as the basis to define infection stages as healthy, slightly infected and severely infected stages corresponding to the healthy thallus, area beyond the lesion in infected thallus and the lesion area, respectively.

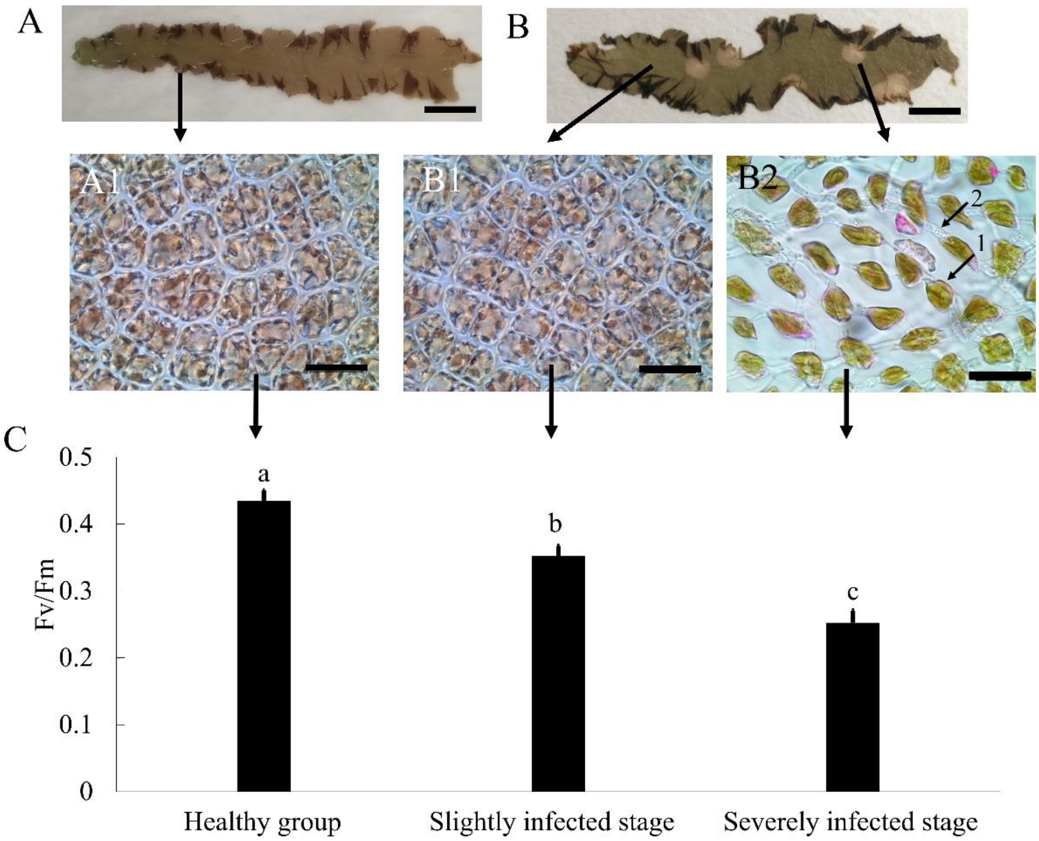

Fig S7. Histopathological features and maximum PSII quantum yield of *Pyr. yezoensis* infected with *Pyt. porphyrae*

A: Healthy *Pyr. yezoensis* thallus of the control group. A1: Microscopy images of healthy *Pyr. yezoensis* thallus. B: Infected *Pyr. yezoensis* thalli with a lesion. B1: Microscopy images of the area beyond the lesion on infected *Pyr. yezoensis* thallus. B2: Microscope images of lesion on infected

Pyr. yezoensis thallus. C: Fv/Fm(maximal quantum yield of PSII) in different infected stage. Arrow 1 in B2: Infected *Pyr. yezoensis* cell; Arrow 2 in B2: *Pyt. porphyrae* hyphae. Bars in A and B: 1cm; Bars in A1, B1and B2: 50 μm. Different letters in C means significant difference ($P< 0.05$) among three groups.
