# Supplementary material for: Transcriptomic Insights into Innate Immunity Responding to Red Rot Disease in Red Alga Pyropia yezoensis
Source: Int J Mol Sci. 2019 Nov 27;20(23):5970. doi: 10.3390/ijms20235970 (PMC6928737; doi:10.3390/ijms20235970)
Supplement: Supplementary file 1 [file ijms-20-05970-s001.zip › TableS1.pdf]

Table S1. Proteins sequences used in phylogenetic analysis

**Proteins**	**Species**	**GenBank accession number**
C-type lectin	*Arabidopsis thaliana*	NP_175641.1
	Arabidopsis thaliana	OAP12328.1
	Oryza sativa	XP_015617554.1
	Oryza sativa	XP_015617555.1
	Nannoshloropsis gaditana	EWM26794.1
	Nannoshloropsis gaditana	EWM26828.1
	Chlamydomonas reinhardtii	PNW77785.1
	Chlamydomonas reinhardtii	PNW73414.1
	Chlamydomonas reinhardtii	PNW75982.1
	Porphyra umbilicalis	OSX81057.1
	Porphyra umbilicalis	OSX70860.1
	Porphyra umbilicalis	OSX80033.1
L-type lectin	*Arabidopsis thaliana*	NC_003076.8
	Arabidopsis thaliana	NC_003074.8
	Oryza sativa	NC_029264.1
	Oryza sativa	NC_029263.1
	Chondrus crispus	XP_005718310.1
	Porphyra umbilicalis	OSX69288.1
	Porphyra umbilicalis	OSX69364.1
	Ectocarpus siliculosus	Esi0227_0052.1
	Ectocarpus siliculosus	Esi0225_0050.1
	Ectocarpus siliculosus	Esi0000_0111.1
R proteins	*Arabidopsis thaliana*	NP_193428.2
	Arabidopsis thaliana	NP_001031652.1
	Chromochloris zofingiensis	UNPLg00039.t1
	Chromochloris zofingiensis	UNPLg00131.t1
	Chromochloris zofingiensis	UNPLg00134.t1
	Chromochloris zofingiensis	UNPLg00127.t1
	Porphyra umbilicalis	OSX80423.1
	Ectocarpus siliculosus	Esi0015_0171.1
	Ectocarpus siliculosus	Esi0104_0065.1
	Porphyra umbilicalis	OSX80000.1
	Ectocarpus siliculosus	Esi0000_0412.1
	Porphyra umbilicalis	OSX80299.1
	Porphyra umbilicalis	OSX79821.1
	Ectocarpus siliculosus	Esi0000_0090.1
	Porphyra umbilicalis	OSX80057.1
	Porphyra umbilicalis	OSX77688.1
RBOH	*Arabidopsis thaliana*	NC_003076.8

	Arabidopsis thaliana	NC_003070.9
	Zea mays	NC_024461.2
	Oryza sativa	NC_029259.1
	Chlamydomonas reinhardtii	XM_001691803.1
	Chlamydomonas reinhardtii	XM_001691804.1
	Chondrus crispus	XP_005718545.1
	Porphyra umbilicalis	OSX69091.1
	Porphyra umbilicalis	OSX73467.1
	Phaeodactylum tricornutum	XP_002179812.1
Cellulase	*Arabidopsis thaliana*	NM_001036005.2
	Arabidopsis thaliana	NC_003076.8
	Oryza sativa	KF214526.1
	Zea mays	NM_001138649.1
	Zea mays	XM_008680545.2
	Monoraphidium neglectum	XM_014049505.1
	Micromonas commoda	XM_002501438.1
	Chondrus crispus	XP_005717153.1
	Gracilariopsis chorda	PXF45766.1
Protease inhibitor	*Arabidopsis thaliana*	NC_003070.9
	Oryza sativa	NC_029257.1
	Oryza sativa	NC_029261.1
	Chromochloris zofingiensis	NW_001843709.1
	Arabidopsis thaliana	NC_003076.8
	Chlorella variabilis	NW_005435085.1
	Volvox carteri	NW0033077870.1
	Chondrus crispus	XP_005717409.1
	Porphyra umbilicalis	OSX79466.1
	Porphyra umbilicalis	OSX71446.1
