# Supplementary figures and images for: Transcriptomic Insights into Innate Immunity Responding to Red Rot Disease in Red Alga Pyropia yezoensis
Source: Int J Mol Sci. 2019 Nov 27;20(23):5970. doi: 10.3390/ijms20235970 (PMC6928737; doi:10.3390/ijms20235970)

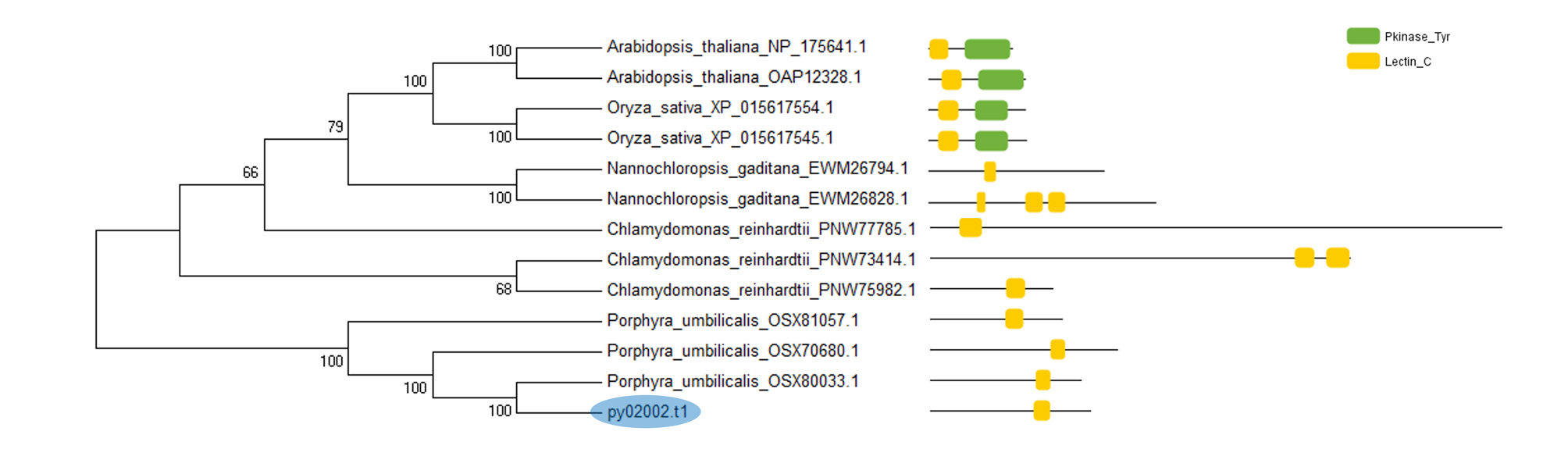

Supplement: Supplementary file 1 [file ijms-20-05970-s001.zip › FIG S1.tif]

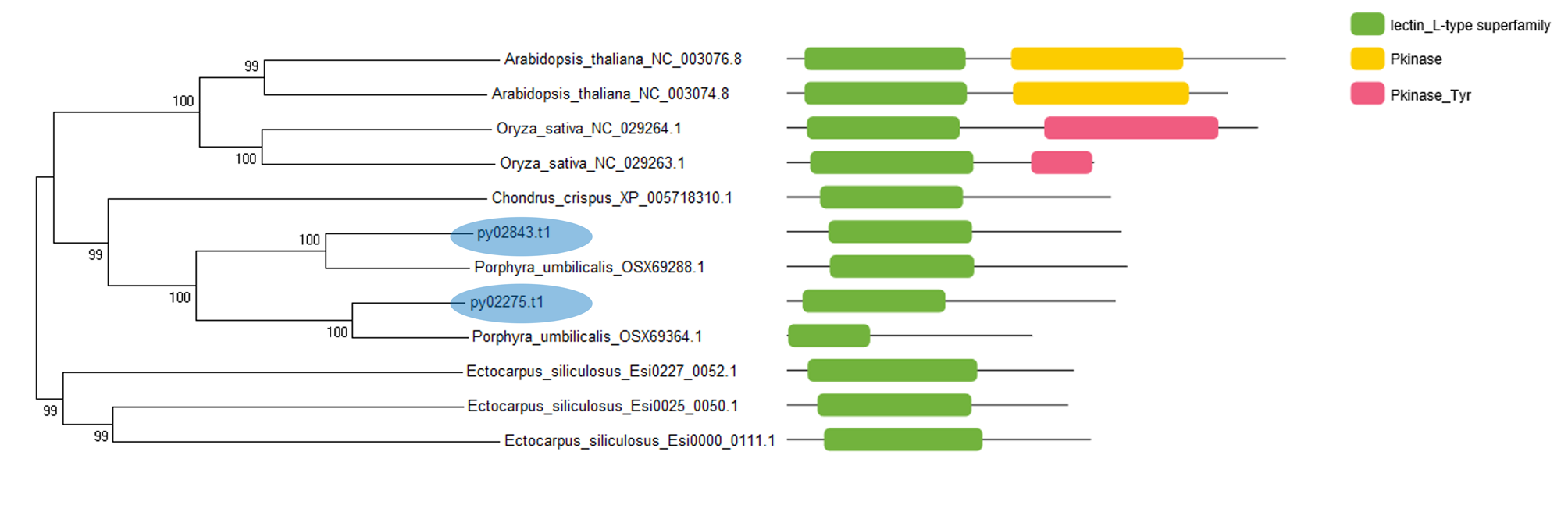

Supplement: Supplementary file 1 [file ijms-20-05970-s001.zip › FIG S2.tif]

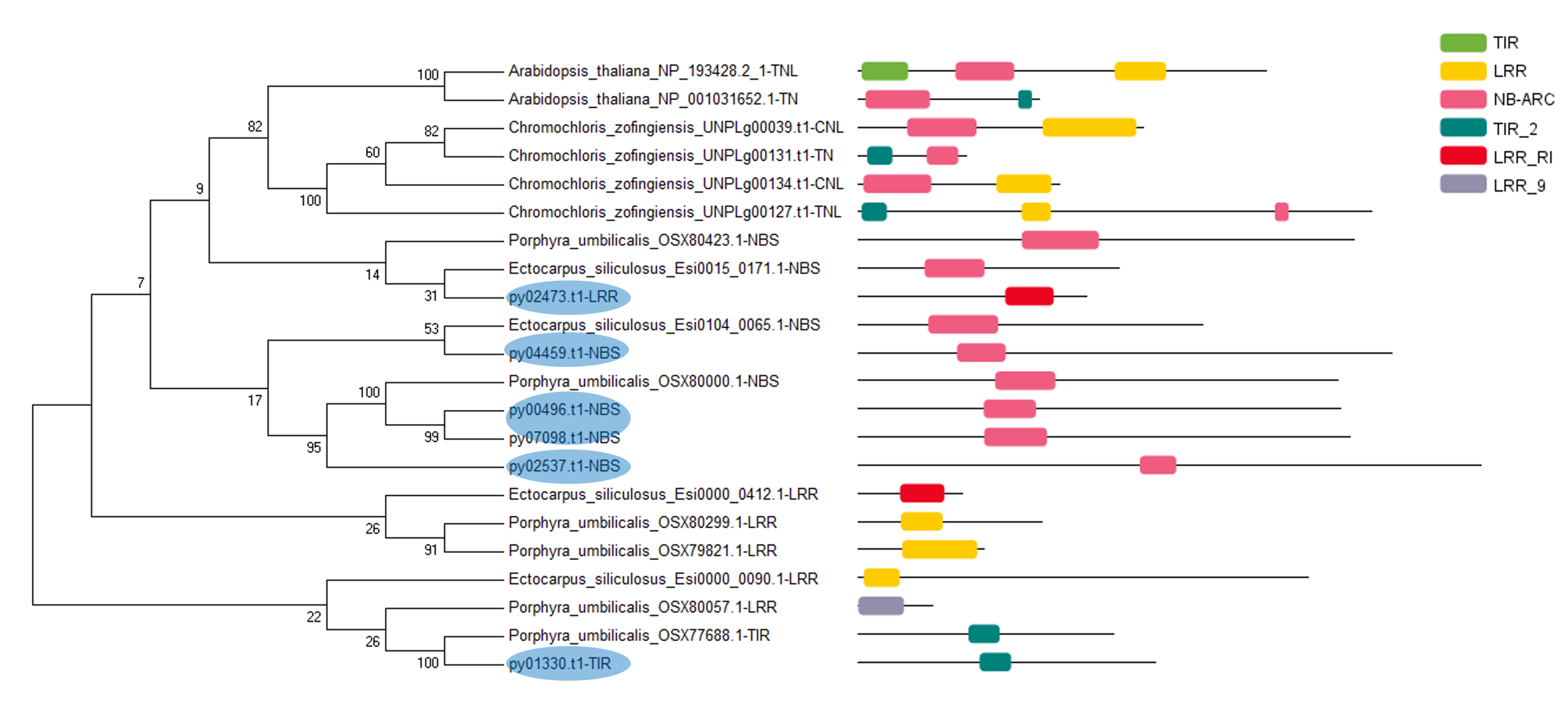

Supplement: Supplementary file 1 [file ijms-20-05970-s001.zip › FIG S3.tif]

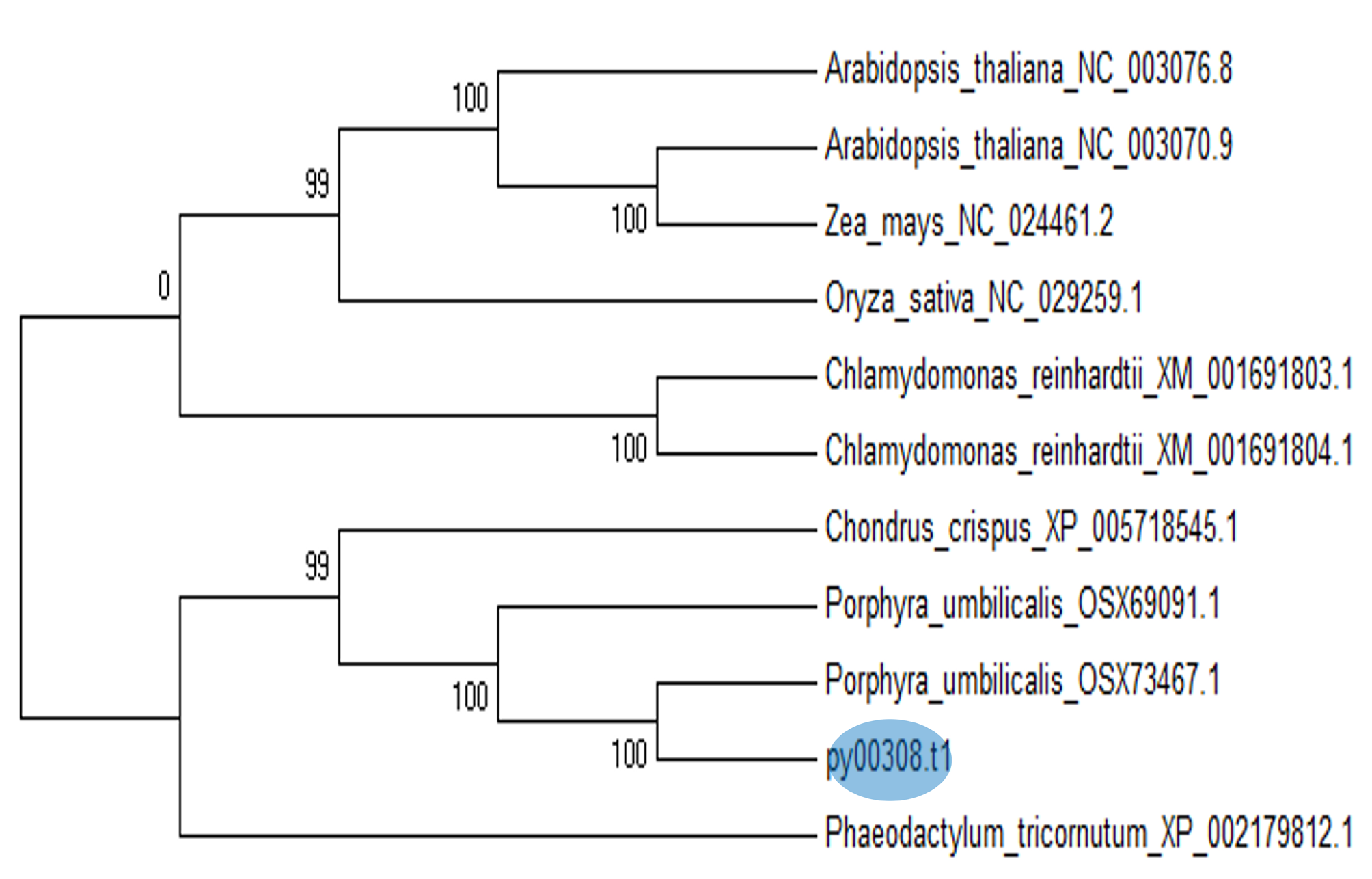

Supplement: Supplementary file 1 [file ijms-20-05970-s001.zip › FIG S4.tif]

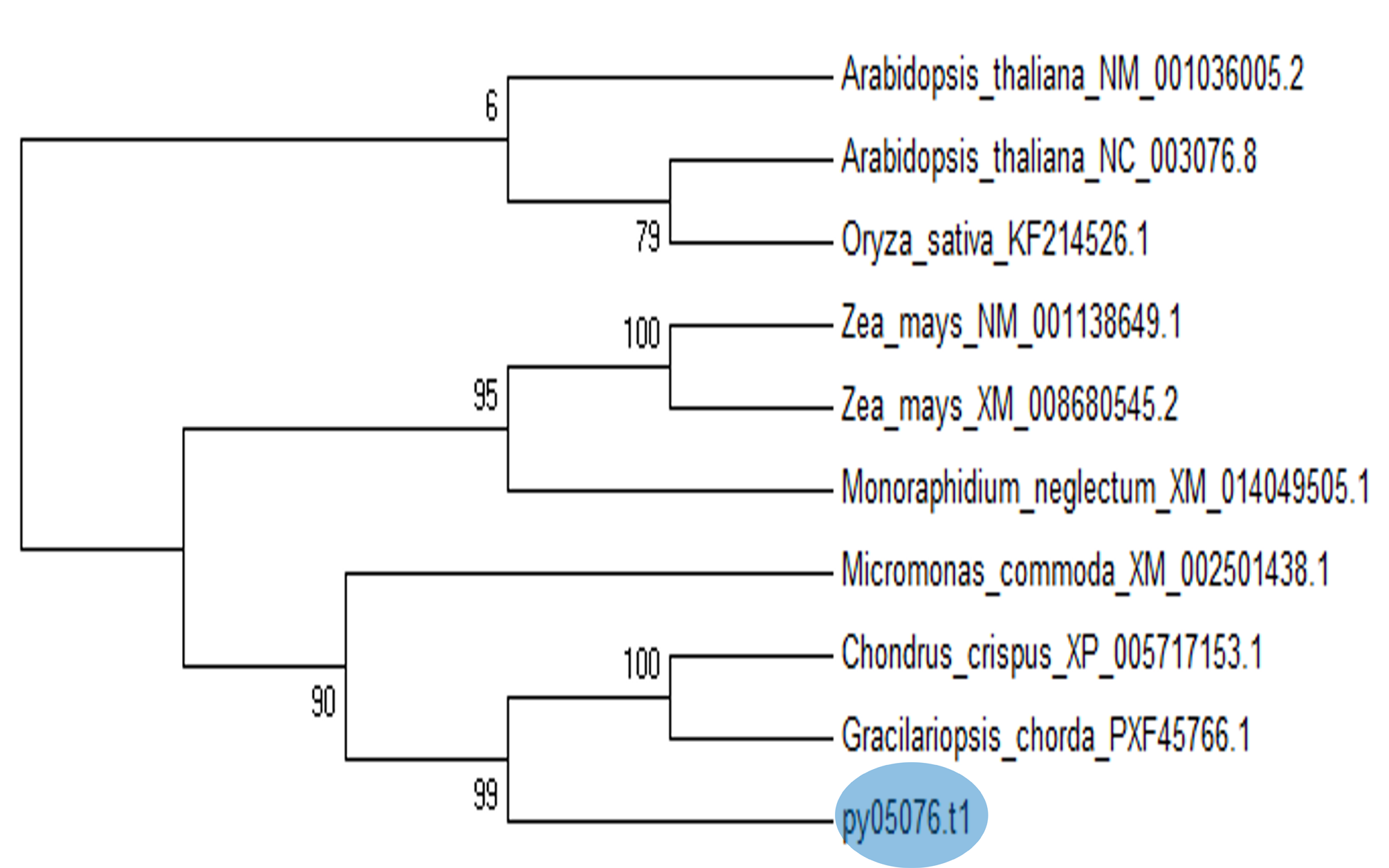

Supplement: Supplementary file 1 [file ijms-20-05970-s001.zip › FIG S5.tif]

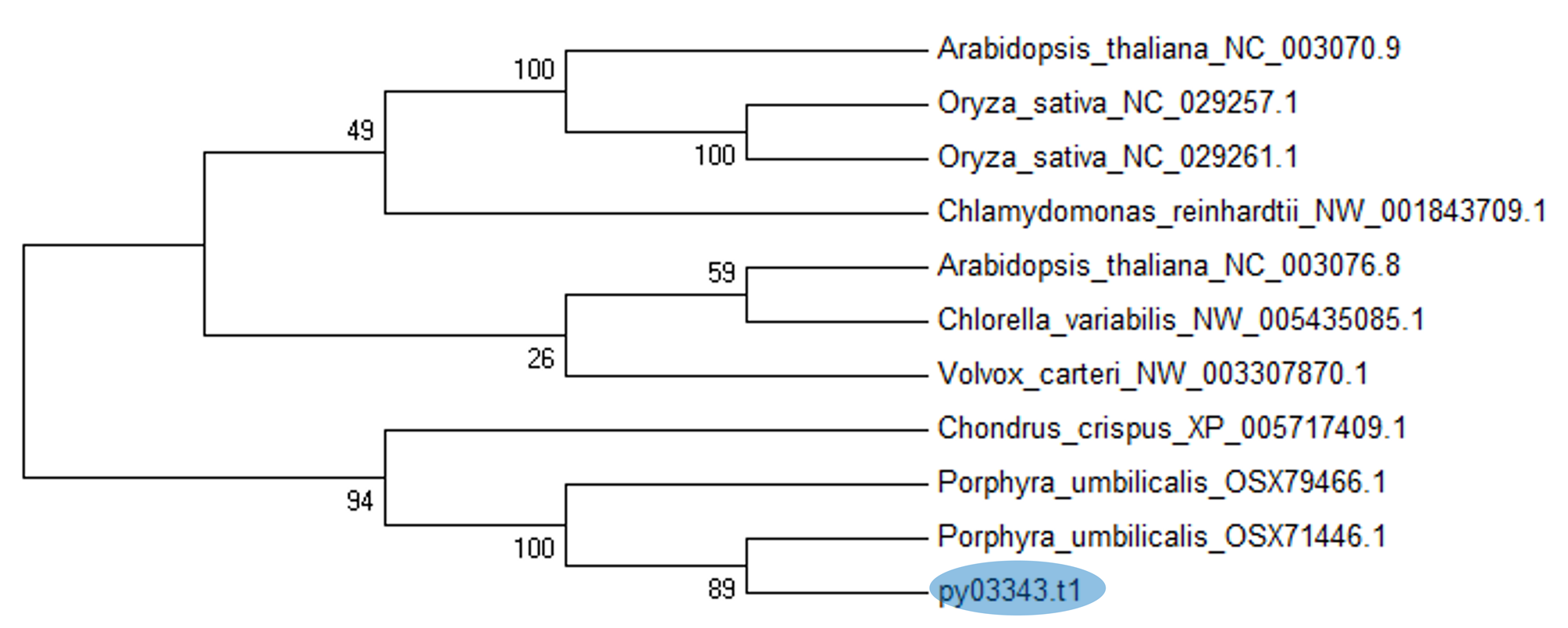

Supplement: Supplementary file 1 [file ijms-20-05970-s001.zip › FIG S6.tif]

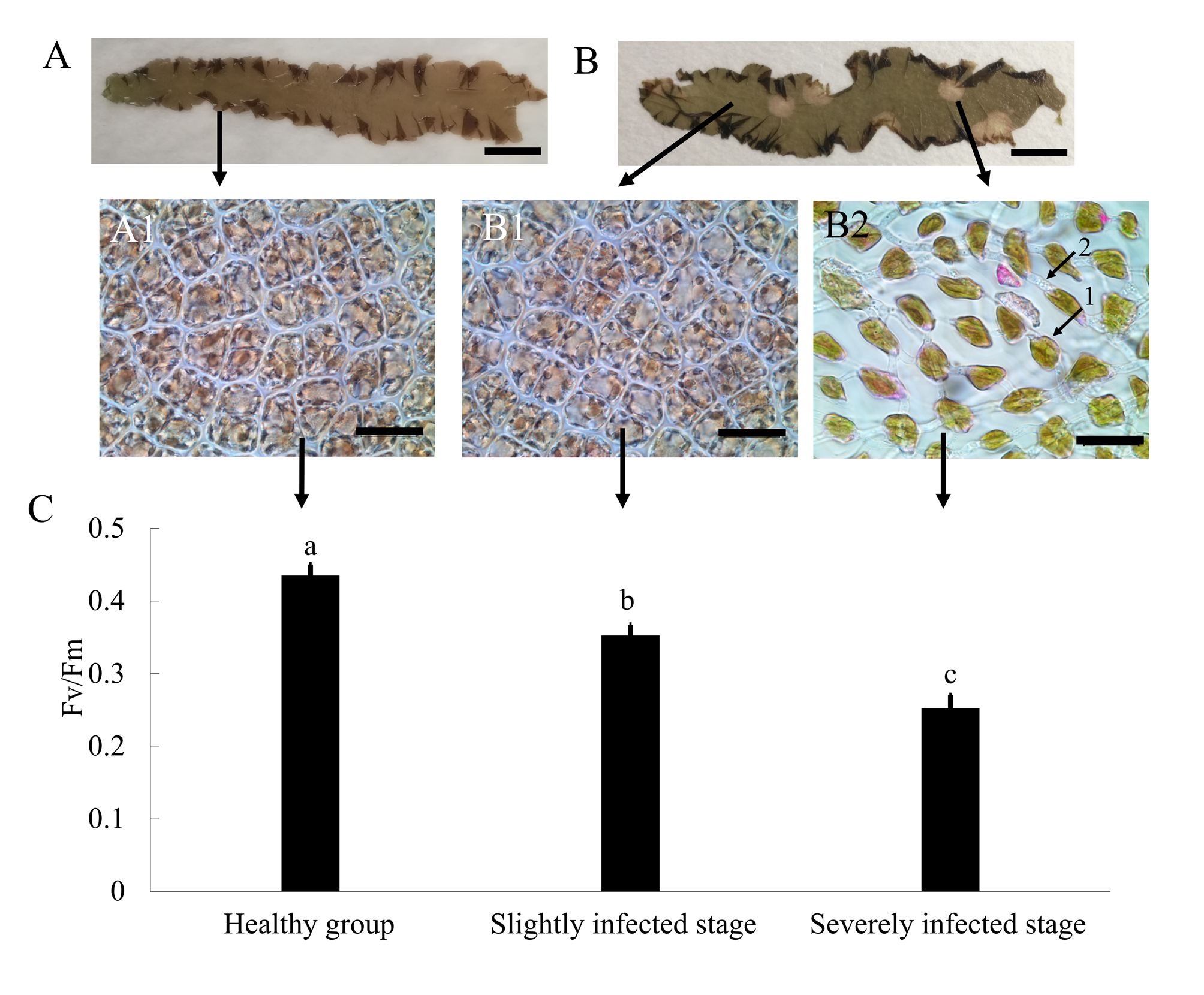

Supplement: Supplementary file 1 [file ijms-20-05970-s001.zip › FIG S7.tif]
